# Exoskeleton-assisted gait training to improve gait in individuals with spinal cord injury: a pilot randomized study

**DOI:** 10.1186/s40814-018-0247-y

**Published:** 2018-03-05

**Authors:** Shuo-Hsiu Chang, Taimoor Afzal, TIRR SCI Clinical Exoskeleton Group, Marcie Kern, Chris White, Hannah Trammell, Marie Beirne, Matthew Davis, Jeffrey Berliner, Gerard E. Francisco

**Affiliations:** 1 Department of Physical Medicine and Rehabilitation, the University of Texas Health Science Center at Houston, 1333 Moursund Street, Houston, TX 77030 USA; 2 Center for Wearable Exoskeletons, NeuroRecovery Research Center at TIRR Memorial Hermann, 1333 Moursund Street, Houston, TX 77030 USA; 3 TIRR Memorial Hermann, 1333 Moursund Street, Houston, TX 77030 USA; 4 Craig Hospital, 3425 S Clarkson St, Englewood, CO 80113 USA

**Keywords:** Rehabilitation, Exoskeleton, Spinal cord injury

## Abstract

**Background:**

Robotic wearable exoskeletons have been utilized as a gait training device in persons with spinal cord injury. This pilot study investigated the feasibility of offering exoskeleton-assisted gait training (EGT) on gait in individuals with incomplete spinal cord injury (iSCI) in preparation for a phase III RCT. The objective was to assess treatment reliability and potential efficacy of EGT and conventional physical therapy (CPT).

**Methods:**

Forty-four individuals were screened, and 13 were eligible to participate in the study. Nine participants consented and were randomly assigned to receive either EGT or CPT with focus on gait. Subjects received EGT or CPT, five sessions a week (1 h/session daily) for 3 weeks. American Spinal Injury Association (ASIA) Lower Extremity Motor Score (LEMS), 10-Meter Walk Test (10MWT), 6-Minute Walk Test (6MWT), Timed Up and Go (TUG) test, and gait characteristics including stride and step length, cadence and stance, and swing phase durations were assessed at the pre- and immediate post- training. Mean difference estimates with 95% confidence intervals were used to analyze the differences.

**Results:**

After training, improvement was observed in the 6MWT for the EGT group. The CPT group showed significant improvement in the TUG test. Both the EGT and the CPT groups showed significant increase in the right step length. EGT group also showed improvement in the stride length.

**Conclusion:**

EGT could be applied to individuals with iSCI to facilitate gait recovery. The subjects were able to tolerate the treatment; however, exoskeleton size range may be a limiting factor in recruiting larger cohort of patients. Future studies with larger sample size are needed to investigate the effectiveness and efficacy of exoskeleton-assisted gait training as single gait training and combined with other gait training strategies.

**Trial registration:**

Clinicaltrials.org, NCT03011099, retrospectively registered on January 3, 2017.

## Background

Inability to walk is one of the major consequences of spinal cord injury (SCI). As of the year 2010, 265,000 people have sustained a SCI in the USA with nearly 61% having incomplete spinal lesions [1]. As a result of SCI, individuals may experience a loss of independence in mobility impacting their community participation and integration and leading to a decreased quality of life [2]. In order to restore the capability for locomotion in a short period of inpatient stay and outpatient therapy, gait rehabilitation usually focuses solely on providing compensatory strategies such as walking with assistive device and braces [3]. However, compensatory strategies limit the potential regeneration and reintegration of the neuromuscular system that leads to functional recovery. Thus, the current trend of SCI rehabilitation has emphasized more on task-dependent or activity-dependent neuromusculoskeletal plasticity and recovery [4].

Neuroplasticity, a condition and ability of modification of neural pathways and synapses in the nervous system, plays a critical role in motor and functional recovery [5]. A substantial number of research studies have demonstrated that neural plasticity and cortical reorganization could occur through systematic execution of task-specific training which leads to recovery of walking after sustaining neurological injury [6–11]. One of the most commonly seen examples of task-specific training for gait is body weight-supported treadmill training (BWSTT). BWSTT produces a large number of stepping repetitions that could induce neuroplastic changes at both the spinal and the cortical levels [12] and lead to gait improvement [13]; however, the results are inconclusive. Several studies involving BWSTT have suggested to be beneficial, but the improvements were not significantly different when compared to conventional overground walking gait [14, 15]. A study by Duncan et al. showed no superiority of BWSTT over home-based physical therapy in improving the functional level of walking and reducing the incidence of falls in individuals with stroke [14]. These findings could be explained, partly by the difference in gait kinematics and subject involvement between treadmill and overground walking. Some gait kinematics when walking on a treadmill are not an exact replica to overground gait kinematics as the body does not move forward in space over the lower limbs [16, 17]. It is also difficult to judge the patient’s active involvement which is a key factor in administering effective rehabilitation [12]. To have more effective gait training that follows task-specific training principles, overground gait training with body weight support features from exoskeleton robotic devices should be considered.

Lower limb robotic assistive devices known as exoskeletons have been developed to assist individuals with lower limb paralysis and weakness to walk [18] and can be used as a gait training device [19]. Exoskeleton robotic devices utilize the user’s movements to control externally powered gait. Therefore, the users of the exoskeleton must be both physically and cognitively engaged to advance the limb. The device is equipped with computer-controlled motors at the hip, knee, and even ankle joints to provide assistance in sit-to-stand, stand-to-sit, upright standing, and walking tasks [18]. The developments have been inspired by the recent advances in technology, with a variety of them being utilized in rehabilitation centers to assist individuals with disability. In recent studies, the safety of walking with wearable exoskeleton devices has been clinically evaluated [18–20], and it is safe for individuals with thoracic SCI, when utilized in a controlled environment with assistance by a trained personnel. Compared to BWSTT, exoskeleton-assisted training could provide potential advantages such as the ability to provide overground walking by using a biomechanical reciprocating pattern that allows achievement of hip extension and full loading of the lower limbs that is similar to a natural gait pattern and engages active involvement. Moreover, the reciprocal gait feature that provides the capability of producing repeated patterns of walking could facilitate neural plasticity. However, the effectiveness of exoskeleton-assisted gait training for individuals with SCI remains unclear.

The primary purpose of this pilot study was to investigate the feasibility of exoskeleton-assisted gait training (EGT) in individuals with chronic incomplete SCI (iSCI). The primary feasibility objectives were assessing patient eligibility, pre-assessment, randomization process, treatment reliability, and post-assessment. The secondary objective of the study was to investigate the potential efficacy of EGT on motor and gait performance compared to conventional physical therapy (CPT) gait training. We hypothesized that subjects who received EGT would show greater improvement in motor and gait performance compared to those who received CPT designed for gait training. Studies that have included complete SCI populations have examined the clinical effectiveness, feasibility, and safety during exoskeleton-assisted walking [18, 21]. As the focus of this study was to examine the potential efficacy of training with exoskeleton on gait and potential underlying mechanisms that lead to improvement, therefore, we only considered incomplete SCI individuals who were also ambulatory. Moreover, chronic individuals particularly > 6 months post-injury reach a stable level of recovery, and any observed improvement from any intervention could be attributed to the intervention itself, unlike acute and sub-acute stage where the observed improvements could also be a result of spontaneous recovery [22]. Further, acute and sub-acute SCI may still have other medical complications that exclude them from utilizing wearable exoskeletons. The results of this study will allow us to ascertain if the complete procedure, i.e., assessing patient eligibility, baseline assessment, randomization process, treatment reliability, and post-assessments, could be conducted in a well-defined manner prior to a definitive randomized controlled trial (RCT).

## Methods

### Design, setting, and subjects

We conducted a parallel-group randomized controlled pilot trial (Houston, TX, USA 2014-2016). Between January 2014 and March 2015, clinical staff at TIRR Memorial Hermann hospital locations in Houston, Texas, scanned and identified potential participants that met the basic study inclusion criteria. Later, the interested participants were screened over the phone by a member of the research staff. If the participants passed the phone screen, they were invited for an onsite screening. The onsite screening was performed by a member of the research team who also consented them. All participants gave their written consent to participate in the study. The study protocol was approved by the Committee for Protection of Human Subjects (CPHS) at the University of Texas Health Science Center at Houston.

Individuals with confirmed diagnosis of chronic motor iSCI classified by the American Spinal Injury Association Impairment Scale (AIS) grades C or D, above the T12 level, were recruited to participate in the study. Inclusion criteria were 18 years of age or older, male or non-pregnant female, at least 6 months after injury, height between 1.5 and 1.88 m, weight less than 100 kg, able to independently stand for 2 min with or without an assistive device and with or without orthoses distal to the knee, and able to follow three-step instructions for cognitive assessment. Subjects were excluded if they had any of the following: presence of clinical signs of lower motor neuron injury; history of severe neurologic injuries other than SCI (multiple sclerosis, cerebral palsy, amyotrophic lateral sclerosis, traumatic brain injury, cerebrovascular accident, etc.); severe comorbidities such as active infections, heart, lung, or circulatory conditions, pressure ulcers, or any skin issues that would prevent wearing the device; documented severe osteoporosis affecting the hip and spine; severe spasticity in the lower extremities (Modified Ashworth ≥ 3) or uncontrolled clonus; unstable spine; unhealed limb or pelvic fractures; range of motion restrictions that would prevent a subject from achieving a normal, reciprocal gait pattern or would restrict a subject from completing normal sit-to-stand or stand-to-sit transitions; upper extremity strength deficits that limit ability to support and balance on a front rolling walker or crutches; heterotopic ossification that resists functional range of motion in lower extremities; contractures (> 15.0° at the hips or > 20.0° at the knees); psychiatric or cognitive comorbidities resulting in motor planning or impulsivity concerns; colostomy; or received any physical therapy intervention within 3 months prior to enrollment in the study.

### Randomization and training protocol

The subjects were randomized by the members of the research team into two groups (EGT and CPT) by drawing lots, maintaining allocation concealment. However, it was impossible to blind the subjects to their allocation as EGT and CPT are completely different interventions requiring extensive subject involvement in training. Exoskeleton utilized in this study was Ekso® (Ekso Bionics, Richmond, CA). In EGT group, subjects donned the Ekso exoskeleton and participated in individualized treatment sessions that included sit to stand, static and dynamic standing balance, weight shifting, walking, turning, and stand to sit. Each training session was 60 min long. The time required to don and doff the device was not included in the training time. The training was held 5 days per week for 3 weeks with a total of 15 sessions. During the training period, the subjects were required to maintain the same amount and level of regular daily physical activity and exercise.

The CPT group received physical therapy designed to facilitate gait improvement. This included individualized treatment sessions consisting of stretching, strengthening, balance training, standing, sit to stand, stair, and gait training. Subjects were not allowed to participate in any form of robotic-assisted or body weight-supported treadmill training during the study period. Each training session lasted 60 min, and the training was held for a total of 15 sessions with 5 days per week for 3 weeks. Consistent with the EGT group, subjects were required to maintain the same amount and level of regular daily physical activity and exercise during the study period.

### Feasibility objectives

We evaluated four primary objectives to assess the feasibility of conducting the EGT protocol.

Patient eligibility: We assessed the patient eligibility by determining the percentage of patients who were eligible for the study and the percentage who were ineligible due to the exo size range limitations.

Outcome assessments (pre and post): The feasibility of the outcome assessment sessions was assessed by determining the number of assessment parameters that the subjects were able to complete.

Treatment reliability: We assessed the treatment reliability by determining the number of adverse events occurring during the trainings or the assessments.

### Assessment protocol

Pre- and post-assessment sessions were conducted at the pre- and immediate post-training for both groups by a physical therapist who was masked of the group assignment. The pre-training session included an initial evaluation of each subject’s height, weight, range of motion, sensation, ability to walk, and muscle strength. The following measures were assessed during both the pre- and post-assessment.

#### Lower Extremity Motor Score

The ability for an individual to volitionally contract muscles in accordance with myotomes is assessed using the American Spinal Injury Association (ASIA) Lower Extremity Motor Score (LEMS). Strength is graded on a scale of 0–5. A thorough description of the assessment procedure is specified in [23].

#### Gait spatial and temporal characteristics

Spatial and temporal characteristics of gait were measured using the GAITRite system (GAITRite, CIR system Inc., USA, 2008). The standard GAITRite walkway contained six sensor pads encapsulated in a rolled up carpet having an active area of 3.66 m in length and 0.61 m in width [24]. During the walk over the walkway, footfalls were captured by the sensors as a function of time. The information was stored and analyzed offline for footfall patterns. The parameters evaluated were cadence, step length, stride length, and stance and swing phase durations, and the mean of the three repetitions was used [25].

#### 10-Meter Walk Test

Gait speed was assessed with the 10-Meter Walk Test (10MWT). The subjects walked for 14 m to account for potential acceleration and deceleration effects. The time was recorded after the subject walked 2 m and was stopped 2 m before the end line. Subjects were instructed to maintain a comfortable pace and walk in a straight line over the required distance. The subjects also had the option to use a preferred assistive device, including minimal physical assistance as needed. The speed was measured in meters per second (m/s).

#### 6-Minute Walk Test

Gait endurance was assessed using the 6-Minute Walk Test (6MWT) [26]. Subjects walked for 6 min at their self-selected speed and could rest when they felt unable to continue. The total walking distance was recorded. Use of any physical assistive device or bracing was documented. Each subject used the same assistive device or bracing at all assessment sessions. The distance covered during the 6MWT is measured in meters (m).

#### Timed Up and Go

Multi-task mobility including sit-to-stand transfers and balance were assessed using the Timed Up and Go (TUG) test [27]. The time was recorded when the subjects rise from the chair and was stopped when the subjects sit on it. In between, the subjects walk 3 m, turn around, and again walk 3 m. The time was measured in seconds (s).

10MWT, 6MWT, and TUG are commonly and widely used functional ambulation outcome measures and have good test-retest, inter-observer reliability, and construct validity in ambulatory SCI [28]. As the focus of this study is on ambulatory SCI, therefore, we proposed using 10MWT, 6MWT, and TUG as the assessment measures.

### Statistical analysis

As this was a pilot study, sample size calculation was not performed [29]. The aim was to recruit at least 10 subjects (five in each intervention) as this would be a large enough sample to infer about the practicality of delivering the interventions in iSCI population, randomization process, treatment reliability, and assessments. Descriptive analysis and mean difference estimated with 95% confidence intervals (CI) were performed to examine within-group differences.

## Results

**Fig. 1:**
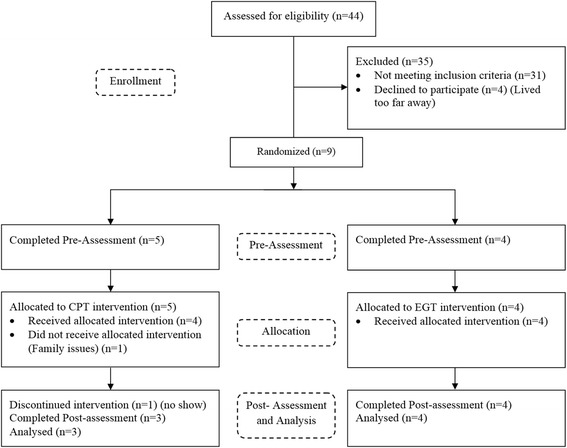
Consolidated Standards of Reporting Trials (CONSORT) diagram. CONSORT showing the enrollment process for the iSCI subjects that were included or excluded in the study

Forty-four subjects were screened for the study, 13 were eligible and nine of the 13 subjects consented to participate. Two participants withdrew during training, and seven participants completed the study. The Consolidated Standards of Reporting Trials (CONSORT) diagram is shown in Fig. 1. The mean age of subjects in EGT (1 female, 3 males) was 56 years (SD 17 years) with a post-injury duration of 15 years (SD 14 years), and the mean age of subjects in CPT (1 female, 2 males) was 60 years (SD: 2 years) with a mean post-injury duration of 7 years (SD 3 years). The level of lesion ranged from C4 to T12 in the EGT and C5 to C12 in the CPT. Subject characteristics are shown in Table 1.

**Table 1:** Participant characteristics

Characteristics	EGT					CPT			
	E1	E2	E3	E4	Mean (SD)	C1	C2	C3	Mean (SD)
Age	30	66	62	64	56 (17)	59	63	59	60 (2)
Weight (kg)	83	72	81	78	79 (5)	92	81	55	76 (19)
Height (m)	1.75	1.65	1.72	1.78	1.7 (0.1)	1.78	1.83	1.6	1.7 (0.1)
Gender	M	F	M	M		M	M	F	
Injury level	C7	T12	T12	C4		T12	C5	T12	
AIS classification	C	D	D	D		C	D	D	
Years post-injury	2	6	16	34	15 (14)	5	6	10	7 (3)

### Feasibility objectives

Thirteen subjects were eligible to participate in the study from a total of 44 participants (30% success rate). Of the 31 screen failures, two subjects did not meet the height and weight requirements, whereas one subject met the height requirement but had long tibia and femur bones. Therefore, 10% of the screen failures were a result of the subjects not meeting the inclusion criteria for height and weight. All subjects were able to complete assessment protocols during the pre- and post-assessment sessions. Regarding the treatment reliability, in the CPT group, all subjects completed the assessment and training sessions without the occurrence of an adverse event. In the EGT group, only one subject acquired ankle soreness after a training session. However, the subject was able to continue the study protocol once the ankle soreness disappeared.

### ASIA LEMS

**Fig. 2:**
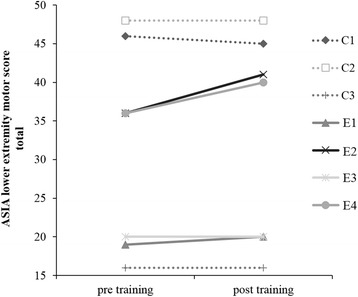
Total AISA lower extremity motor score pre- and post-training. EGT subjects showed an increase in the total LEMS score. No significant increase was observed in percentage change during post-assessment compared to pre-assessment. Dotted lines represent the CPT group. Solid lines represent the EGT group

ASIA lower extremity motor scores at the pre- and post-training for each participant are shown in Fig. 2. Subjects E1, E2, and E4 showed improvement in LEMS score, but no change in LEMS score was observed in subject E3.

### Gait characteristics

**Fig. 3:**
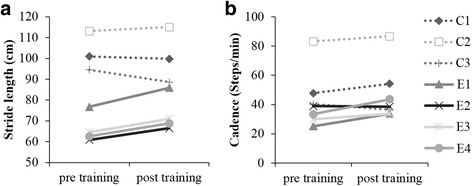
Pre- and post-assessment values for **a** stride length and **b** cadence in EGT and CPT subjects. EGT groups showed increased stride length. CPT group showed a decrease in stride length. Dotted lines represent the CPT group. Solid lines represent the EGT group

Increase in the stride length was found in the EGT group (pre-stride length 66 cm (SD 7 cm), post-stride length 72 cm (SD 9 cm). The difference between pre- and post-stride length was statistically significant (mean difference 6.33; 95% CI = (3.4, 9.3)). However, no significant change in stride length was observed in the CPT group (pre-stride length 102 cm (SD 11 cm), post-stride length 99 cm (SD 15 cm), mean difference − 2.2; 95% CI = (− 9.9, 5.5)). All subjects in the EGT group displayed increase in stride length (Fig. 3a).

Increase in cadence was observed in the EGT group, increasing from 32 steps/min (SD 6 steps/min) during pre-assessment to 37 steps/min (SD 5 steps/min) during post-assessment; however, the difference was not significant (mean difference 5.6; 95% CI = (− 2.0, 13.1)). The CPT group showed steps/min increase from 57 (SD 23) to 59 (SD 26). The results are shown in Fig. 3b.

The right step length in the EGT group increased (pre-right step length 36 cm (SD 2 cm), post-right step length 40 cm (SD 4 cm)), with a statistically significant difference (mean difference 2.0; 95% CI = (0.7, 8.0)). A significant change was also observed in the right step length in the CPT group (pre-right step length 52 cm (SD 7 cm), post-right step length 53 cm (SD 7 cm)), mean difference 1.2; 95% CI = (0.3, 2.1)). No significant group difference was found in swing phase percentage, stance phase percentage, and left step length in EGT and CPT subjects. These results are shown in Table 2.

**Table 2:** Results of swing phase percentage, stance phase percentage, and step length for EGT and CPT participants

Parameter	EGT			CPT		
	Pre-mean (SD)	Post-mean (SD)	Mean difference (95% CI)	Pre-mean (SD)	Post-mean (SD)	Mean difference (95% CI)
Left						
Swing percentage	16.5 (7.6)	18.3 (7.5)	1.8 (− 5.8, 9.4)	24.5 (8.4)	26.0 (9.0)	2.4 (− 0.3, 3.4)
Stance percentage	83.5 (7.6)	81.7 (7.5)	−1.8 (− 9.4, 5.8)	75.5 (8.4)	74.0 (9.0)	− 2.3 (− 3.4, 0.3)
Step length (cm)	30.0 (5.1)	32.0 (7.0)	2.0 (− 1.7, 5.7)	49.4 (6.2)	46.0 (9.6)	4.4 (− 11.9, 5.1)
Right						
Swing percentage	16.5 (4.8)	18.8 (8.6)	1.8 (− 6.5, 11.2)	26.5 (6.6)	25.0 (7.3)	− 1.5 (− 7.0, 4.1)
Stance percentage	83.6 (4.8)	81.2 (8.6)	− 1.8 (− 11.2, 6.5)	73.5 (6.6)	75.0 (7.3)	1.5 (− 4.1, 7.0)
Step length (cm)	36.0 (2.1)	40.4 (3.7)	2.0 (0.7, 8.0)	52.8 (7.0)	53.4 (6.6)	1.2 (0.3, 2.1)

### Functional activities

Gait speed was measured during the 10MWT. The average speed achieved by the EGT group was 0.17 m/s (SD 0.01 m/s) and 0.22 m/s (SD 0.03 m/s) during pre- and post-assessments respectively. However, the mean difference was not statistically significant (mean difference 0.04; 95% CI = (− 0.02, 0.11))*.* The CPT group walked at a speed of 0.51 m/s (SD 0.28 m/s) and 0.55 m/s (SD 0.31 m/s) during pre- and post-assessments respectively. Also, the mean difference was not statistically significant (mean difference 0.04; 95% CI = (− 0.09, 0.17)).

Multi-task mobility was assessed by TUG with time to complete as the assessment parameter. The EGT group required a mean time of 71 s (SD 23 s) to complete the test during pre-assessment and 55 s (SD 8 s) during post-assessment. However, no statistically significant changes were observed in the mean difference between pre- and post-assessments (mean difference − 15.43; 95% CI = (− 47.5, 16.6)). CPT group required a mean time of 37 s (SD 17 s) to complete the test during pre-assessment and 36 s (SD 17 s) during post-assessment. Although the percentage of improvement observed in the CPT group was less compared to that in the EGT group, the mean difference estimate showed a statistically significant reduction in TUG time (mean difference − 1.6; 95% CI = (− 2.6, − 0.6)).

**Fig. 4:**
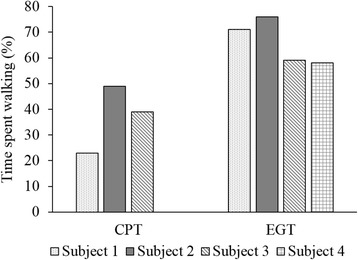
Comparison of walk time during CPT and EGT intervention. Subjects walking with exoskeleton assistance walked more time during the training sessions than CPT subjects

Walking endurance was assessed during 6MWT with distance walked as the assessment parameter. The EGT group walked an average distance of 50 m (SD 23 m) at pre-assessment and 67 m (SD 25 m) at post-assessment. The improvement in 6MWT distance at post-assessment was statistically significant (mean difference 16.9; 95% CI = (1.2, 32.5)). The CPT group walked an average distance of 147 m (SD 87 m) at pre-assessment and 154 m (SD 94 m) at post-assessment. No significant improvement was observed in the 6MWT distance in CPT subjects (mean difference 7.7; 95% CI = (− 9.3, 24.7))*.* The results are also shown in Table 3. Subjects in EGT group spent substantially more time 66% (SD: 9%) on walking compared to subjects in CPT group who spent 37% (SD: 13%) of the time in weight bearing activities (i.e. standing and walking) (Fig. 4).

**Table 3:** Mean of the functional assessment parameters at pre- and post-assessments for EGT and CPT groups and within-group differences

Parameter	EGT			CPT		
	Pre-mean (SD)	Post-mean (SD)	Mean difference (95% CI)	Pre-mean (SD)	Post-mean (SD)	Mean difference (95% CI)
10MWT (m/s)	0.17 (0.01)	0.22 (0.03)	0.04 (− 0.02, 0.11)	0.51 (0.28)	0.55 (0.31)	0.04 (− 0.09, 0.17)
6MWT (m)	50 (23)	67 (25)	16.9 (1.2, 32.5)	147 (87)	154 (94)	7.7 (− 9.3, 24.7)
TUG (s)	71 (23)	55 (8)	− 15.4 (− 47.5, 16.6)	37 (17)	36 (17)	− 1.6 (− 2.6, − 0.6)

## Discussion

To our knowledge, this study is one of the few pilot studies to investigate the feasibility and potential efficacy of exoskeleton-assisted gait training (EGT) and conventional physical therapy (CPT) on gait performance in individuals with iSCI. As individuals with SCI can walk safely with exoskeleton as an assistive device [18, 19] and there is a potential to receive various health benefits such as improved bowel and bladder function and reduced spasticity [18], wearable exoskeletons could also be utilized as a gait training device to facilitate motor and gait function recovery and health promotion in this population. By increasing the duration of the training and inducing large number of the repetitions, the use of exoskeleton provides intense repetitive locomotor training to facilitate gait function recovery as compared to conventional physical therapy gait training. As we observed improvement in both EGT and CPT groups, the effectiveness of EGT requires further investigation. Our experience also indicated that the use of wearable exoskeleton may relieve clinicians (i.e., physical therapists) from improper body mechanics and musculoskeletal burden in manual body weight support and guidance during locomotion training. Although the sample was small and the results are not statistically significant to support a strong conclusion, the results have provided insightful and critical information such as assessing patient eligibility, baseline assessment, randomization process, treatment reliability, and post-assessments could be conducted in a well-defined manner prior to a definitive RCT.

The current protocol was feasible as all subjects were able to complete the training and assessments. The training frequency (five sessions/week) was feasible; however, this may not be ideal for persons who are employed full time or need transportation assistance from family members or friends. The subject inclusion and exclusion criteria was feasible and designed appropriately. The exoskeleton technology is new, and only a few clinical trials were or are being conducted; the inclusion and exclusion criteria was designed and determined for subject safety. The only adverse event (ankle soreness) occurred during the EGT, showing that EGT is safe and the training protocol was tolerable. The assessment sessions were 3–4 h long and were performed in one time slot. The patient was able to complete all tasks in one session that shows the reliability of the assessment protocol. The exoskeleton-assisted walking involves a human-machine contact, particularly the thighs and shanks are wrapped with straps attached to the exoskeleton for an upright posture. These areas could potentially develop redness if pressure is applied for a long time. Therefore, it is important to monitor the skin for any potential adverse effects before and after each training session. In addition, the assessment protocol was designed to look at the different parameters such as muscle strength, endurance, gait speed, etc. Static and dynamic balance assessments could be added in future trials to further evaluate the effectiveness of EGT.

An effective gait requires a multi-factor and system control including the neuromuscular, musculoskeletal, cardiopulmonary, sensory, and cognitive systems. We observed improvement in gait characteristics and gait speed and distance in EGT with significant improvement in the stride length, right step length, and 6MWT. The results are similar with the results in the study by Sale et al. [30]. Three subjects with SCI participated in 20 sessions of mobility training using wearable exoskeleton (Ekso), and improvement was found in walking speed, cadence, step length, and walking distance in 6 min after training. The significant improvement in gait speed and distance could be attributed to the improvement in LEMS. The increases in muscle strength could be associated, at least partially, with the effects on neuroplasticity after EGT. Literature has shown that short-term strength and endurance training, especially at higher gait speeds, improves gait parameters and facilitates neuromuscular output [31, 32]. Therefore, EGT may have led to strengthening of intact neural pathways in the subjects and the improvements in gait characteristics. Moreover, the improvement could also be attributed to the cardiopulmonary and musculoskeletal stress induced during exoskeleton-assisted walking. It has been suggested that cardiorespiratory and metabolic demands of exoskeleton-assisted walking are consistent with activities performed at a moderate intensity in individuals with SCI [33] and the perceived exertion when using a powered exoskeleton for assisted walking could range from mild to somewhat hard [34]. Long-term application of exoskeleton-assisted walking, therefore, could be treated as a cardiopulmonary and endurance training with appropriate assistance setting in the device. In addition, EGT could also provide essential afferent inputs such as proprioceptors responding to hip extension for initiating swing (hip position) and extensor load during stance-to-swing transition (external loading) [10]. It also provides an opportunity for hip extension and external load during overground walking, by predetermined walking speed, and inter-limb and intra-limb coordination and kinematics that could enhance the neural output and modulate neuroplasticity for walking in spinal and/or supra-spinal level [20].

It is important to note that the recovery of gait after SCI depends on the type and level of the injury, post-injury medical care, and rehabilitation intervention. EGT could be treated as a standalone gait training program or as a part of the rehabilitation program depending on the treatment goals, recovery phases, or disability severity of the patients; thus, further investigation is required. The design and control of wearable exoskeleton utilized in this study require the user to use and control assistive device such as walker or canes to maintain standing balance and perform lateral and forward trunk sways for movement (stepping) initiation of the exoskeleton. Therefore, the strategy of exoskeleton-assisted training is different between training with paraplegia and quadriplegia. One of the significant differences is the ability to maintain balance and trigger stepping movement when using wearable exoskeleton; however, this could be compensated by body weight supported harness and assistance from the trainer (i.e., assist the user to perform trunk sway). Depending on the impairment and patient goals, potential therapeutic effects, if any, of exoskeleton-assisted gait training could focus on gait and balance for paraplegia and bowel and bladder function or cardiopulmonary strengthening for quadriplegia.

Some important insights regarding the duration and intensity of the study and the randomization process are provided to facilitate the conduction of future RCTs. Our EGT protocol may not provide sufficient intensity. EGT program was held five times per week for 3 weeks with a total of 15 sessions. Short-term (6–12 weeks) strength and endurance training at higher gait speeds improves gait parameters [31, 32]. In Aach et al.’s study, subjects showed improvement in 10MWT, TUG, and 6MWT after 90 days (five times per week) of body weight support of exoskeleton-assisted treadmill training [35]. In the study by Hornby et al., 6MWT started to improve after 4 weeks of training and continued to increase up to 12 weeks [36]. In future studies, longer training duration should be considered when conducting EGT. Moreover, subjects were randomly assigned to either group by drawing lots, not by injury and functioning level. Subjects in the CPT group had better baseline function in 10MWT, 6MWT, and TUG as compared to EGT group but still not close to the normal values in healthy able-bodied. Persons with low functioning may have difficult time at the beginning of the EGT in learning how to maintain balance and coordinate upper and lower limb movement during walking with exoskeleton, and therefore, less time was spent in walking early during training. Statistical comparison of the CPT and EGT groups was not performed due to the modest sample size and the fact that the baseline scores of the two groups were different (CPT higher functioning than EGT). The subjects were allocated to either intervention after randomization, yet the CPT group has a higher functioning level than EGT. This explains that even if the injury level of the individuals is same, their functional level could be different; therefore, the intergroup differences could be impacted because of baseline functional differences. To reduce the likelihood of biased group assignment, different randomization methods could be used when allocating patients to different interventions. We suggest using stratified randomization for balanced and unbiased allocation of individuals. For future definitive RCT, a stratified scheme would be used. Individuals would be assigned to groups based on the functional level, i.e., low functioning, mid-functioning, and high functioning. Then, within each group, randomization scheme can be separately performed reducing the risk of biased group assignment. Finally, one of the practical challenges observed in this study was the small number of patients (30%) who qualified for the study. This was partly (10% screen failures) due to the size range of the exoskeleton (i.e., body height, weight, hip width, and lower limb segment length) which limits the number of subjects who could be fitted in the exoskeleton and qualify for the trial. Future wearable exoskeletons which may have better capability to fit a wider range of body size and a larger scale of trials to investigate effectiveness will be feasible. Conducting a multi-center trial is another potential solution as greater outreach is possible. Apart from the size range of exoskeleton, other factors such as time, location, and intervention assignment also play a major part in subject recruitment.

## Conclusions

Exoskeleton-assisted gait training for individuals with iSCI could improve gait, but studies with a larger cohort of individuals with iSCI are required to signify improvement. The results suggest that powered exoskeletons not only provide iSCI patients the ability to walk overground but may also be utilized for therapeutic intervention to improve gait function. The plausible mechanisms leading to the observed improvement need further investigation; therefore, further studies are needed to measure plasticity and physiological changes in response to training. Moreover, further investigation in the form of large randomized trials is also required to investigate the cost-effectiveness and dose-response relationship of exoskeleton-assisted gait training as standalone training or combined with other gait training in SCI.

## Abbreviations

10MWT: 10-Meter Walk Test
6MWT: 6-Minute Walk Test
AIS: Association Impairment Scale
ASIA: American Spinal Injury Association
CONSORT: Consolidated Standards of Reporting Trials
CPT: Conventional physical therapy
EGT: Exoskeleton-assisted gait training
iSCI: Incomplete spinal cord injury
LEMS: Lower Extremity Motor Score
TUG: Timed Up and Go

## References

[1] Center NSCI (2012). Spinal cord injury facts and figures at a glance. J Spinal Cord Inj Med.

[2] Anderson KD (2004). Targeting recovery: priorities of the spinal cord-injured population. J Neurotrauma.

[3] Dobkin BH (2004). Strategies for stroke rehabilitation. Lancet Neurol.

[4] Lynskey JV, Belanger A, Jung R (2008). Activity-dependent plasticity in spinal cord injury. J Rehabil Res Dev.

[5] Pascual-Leone A, Freitas C, Oberman L, Horvath JC, Halko M, Eldaief M (2011). Characterizing brain cortical plasticity and network dynamics across the age-span in health and disease with TMS-EEG and TMS-fMRI. Brain Topogr.

[6] Hoffman LR, Field-Fote EC (2007). Cortical reorganization following bimanual training and somatosensory stimulation in cervical spinal cord injury: a case report. Phys Ther.

[7] Green JB, Sora E, Bialy Y, Ricamato A, Thatcher RW (1999). Cortical motor reorganization after paraplegia: an EEG study. Neurology.

[8] Kambi N, Tandon S, Mohammed H, Lazar L, Jain N (2011). Reorganization of the primary motor cortex of adult macaque monkeys after sensory loss resulting from partial spinal cord injuries. J Neurosci.

[9] Girgis J, Merrett D, Kirkland S, Metz GA, Verge V, Fouad K (2007). Reaching training in rats with spinal cord injury promotes plasticity and task specific recovery. Brain.

[10] Behrman AL, Bowden MG, Nair PM (2006). Neuroplasticity after spinal cord injury and training: an emerging paradigm shift in rehabilitation and walking recovery. Phys Ther.

[11] George Hornby T, Straube DS, Kinnaird CR, Holleran CL, Echauz AJ, Rodriguez KS (2011). Importance of specificity, amount, and intensity of locomotor training to improve ambulatory function in patients poststroke. Top Stroke Rehabil.

[12] Dobkin BH, Duncan PW (2012). Should body weight-supported treadmill training and robotic-assistive steppers for Locomotor training trot back to the starting gate?. Neurorehabil Neural Repair.

[13] Lo AC, Triche EW (2008). Improving gait in multiple sclerosis using robot-assisted, body weight supported treadmill training. Neurorehabil Neural Repair.

[14] Duncan PW, Sullivan KJ, Behrman AL, Azen SP, Wu SS, Nadeau SE (2011). Body-weight-supported treadmill rehabilitation after stroke. N Engl J Med.

[15] Nooijen CF, ter Hoeve N, Field-Fote EC (2009). Gait quality is improved by locomotor training in individuals with SCI regardless of training approach. J Neuroeng Rehabil.

[16] Puh U, Baer GD (2009). A comparison of treadmill walking and overground walking in independently ambulant stroke patients: a pilot study. Disabil Rehabil.

[17] Alton F, Baldey L, Caplan S, Morrissey MC (1998). A kinematic comparison of overground and treadmill walking. Clin Biomech (Bristol, Avon).

[18] Zeilig G, Weingarden H, Zwecker M, Dudkiewicz I, Bloch A, Esquenazi A (2012). Safety and tolerance of the ReWalk exoskeleton suit for ambulation by people with complete spinal cord injury: a pilot study. J Spinal Cord Med.

[19] Kolakowsky-Hayner SA, Crew J, Moran S, Shah A (2013). Safety and feasibility of using the EksoTM bionic exoskeleton to aid ambulation after spinal cord injury. J Spine.

[20] Bortole M, Venkatakrishnan A, Zhu F, Moreno JC, Francisco GE, Pons JL (2015). The H2 robotic exoskeleton for gait rehabilitation after stroke: early findings from a clinical study. J Neuroeng Rehabil.

[21] Birch N, Graham J, Priestley T, Heywood C, Sakel M, Gall A (2017). Results of the first interim analysis of the RAPPER II trial in patients with spinal cord injury: ambulation and functional exercise programs in the REX powered walking aid. J Neuroeng Rehabil.

[22] Fleerkotte BM, Koopman B, Buurke JH, van Asseldonk EH, van der Kooij H, Rietman JS (2014). The effect of impedance-controlled robotic gait training on walking ability and quality in individuals with chronic incomplete spinal cord injury: an explorative study. J Neuroeng Rehabil.

[23] Thompson CK, Jayaraman A, Kinnaird C, Hornby TG (2011). Methods to quantify pharmacologically induced alterations in motor function in human incomplete SCI. J Vis Exp.

[24] Lee YJ, Kim JY, Kim SY, Kim KH (2016). The effects of trunk kinesio taping on balance ability and gait function in stroke patients. J Phys Ther Sci.

[25] McDonough AL, Batavia M, Chen FC, Kwon S, Ziai J (2001). The validity and reliability of the GAITRite system’s measurements: a preliminary evaluation. Arch Phys Med Rehabil.

[26] Enright PL (2003). The six-minute walk test. Respir Care.

[27] Podsiadlo D, Richardson S (1991). The timed “Up & Go”: a test of basic functional mobility for frail elderly persons. J Am Geriatr Soc.

[28] Lam T, Noonan VK, Eng JJ, Team SR (2008). A systematic review of functional ambulation outcome measures in spinal cord injury. Spinal Cord.

[29] Lancaster GA, Dodd S, Williamson PR (2004). Design and analysis of pilot studies: recommendations for good practice. J Eval Clin Pract.

[30] Sale P, Russo EF, Russo M, Masiero S, Piccione F, Calabrò RS (2016). Effects on mobility training and de-adaptations in subjects with spinal cord injury due to a wearable robot: a preliminary report. BMC Neurol.

[31] Cadore EL, Pinto RS, Bottaro M, Izquierdo M (2014). Strength and endurance training prescription in healthy and frail elderly. Aging Dis.

[32] Eng JJ, Tang PF (2007). Gait training strategies to optimize walking ability in people with stroke: a synthesis of the evidence. Expert Rev Neurother.

[33] Evans N, Hartigan C, Kandilakis C, Pharo E, Clesson I (2015). Acute cardiorespiratory and metabolic responses during exoskeleton-assisted walking overground among persons with chronic spinal cord injury. Topics Spinal Cord Inj Rehabil.

[34] Kozlowski A, Bryce T, Dijkers M (2015). Time and effort required by persons with spinal cord injury to learn to use a powered exoskeleton for assisted walking. Topics Spinal Cord Inj Rehabil.

[35] Aach M, Cruciger O, Sczesny-Kaiser M, Höffken O, Meindl RC, Tegenthoff M (2014). Voluntary driven exoskeleton as a new tool for rehabilitation in chronic spinal cord injury: a pilot study. Spine J.

[36] Hornby TG, Zemon DH, Campbell D (2005). Robotic-assisted, body-weight-supported treadmill training in individuals following motor incomplete spinal cord injury. Phys Ther.

